# A new phase of China-ASEAN health cooperation: the China-ASEAN Beijing Declaration on Cooperation in Innovation of Health Products and Technologies

**DOI:** 10.1186/s41256-024-00401-x

**Published:** 2025-01-14

**Authors:** Jie Qiao, Siyan Zhan, Minghui Ren, Haijun Wang, Yangmu Huang, Zhenyu Zhang, Yanan Luo, Hui Yin, Zhongwei Jia, Wei Huang, Hong Zhou, Jue Liu, Xiaoyun Liu, Qiudan Sun, Xiaojia Li, Jing Bai, Fangjing Liu, Yihong Liu, Yinzi Jin, Ming Xu, on behalf of China-ASEAN Science and Technology Cooperation Center for Public Health

**Affiliations:** 1 Department of Obstetrics and Gynecology, Center for Reproductive Medicine, Peking University Third Hospital, Beijing, China; 2 Department of Epidemiology and Biostatistics, School of Public Health, Peking University, Beijing, China; 3 China Centre for Health Development Studies, Peking University, Beijing, China; 4 Department of Global Health, School of Public Health, Peking University, Beijing, China; 5 Department of Maternal and Child Health, School of Public Health, Peking University, Beijing, China; 6 Department of Occupational and Environmental Health Sciences, School of Public Health, Peking University, Beijing, China; 7 Office of International Cooperation, Peking University Health Science Center, Beijing, China

**Keywords:** China-ASEAN Cooperation, Health Products and Technologies, Research and development, Accessibility

## Abstract

Utilizing innovative methods to advance the research and development (R&D) of health products and enhance their accessibility has become crucial to achieving universal health coverage, addressing public health emergencies, and promoting population health and wellbeing. However, structural contradictions do exist in the supply and demand of health products in the Association of Southeast Asian Nations (ASEAN). With the joint support of the Ministry of Science and Technology of China, the Ministry of Education, and the China-ASEAN Center, Peking University established the China-ASEAN Science and Technology Cooperation Center for Public Health in April 2023. The Center held the Second Annual Forum on China-ASEAN Cooperation in Public Health on June 26–28, 2024, and the participants reached consensus on launching the China-ASEAN Beijing Declaration on Cooperation in Innovation of Health Products and Technologies, which calls on China and ASEAN countries to carry out the following actions: (1) Establish a more effective, open, and inclusive cooperation mechanism for health product innovation towards a China-ASEAN innovation ecosystem; (2) Enhance R&D capabilities by targeting new technologies, methods and appropriate and affordable health products for key populations and addressing diseases prevalent in China and ASEAN; (3) Establish a China-ASEAN Collaboration Center for Health Product Innovation, coordinate regional development plans, and enhance equitable access to pharmaceutical products at the regional level; (4) Accelerate regulatory harmonization in the region by optimizing and improving regulatory modalities of China and ASEAN Countries; and (5) Strengthen cross-sectoral cooperation to build resilient health system and achieve sustainable development of innovation cooperation. The Declaration will play an active role in regional public health governance and development cooperation, in order to promote the R&D and accessibility of health products in the region and to help achieve faster and more equitable access to health products for a broader population.

## Introduction

Innovation is a central theme in the development of the global health industry. Utilizing innovative methods to advance the research and development (R&D) of health products and enhance their accessibility has become crucial to achieving universal health coverage, addressing public health emergencies, and promoting population health and wellbeing [1]. World Health Organization (WHO) has consistently emphasized the importance of a systematic approach to utilizing innovative methods to promote the R&D and accessibility of health products [2]. This requires continuous investment and effective collaborations throughout the product lifecycle, spanning from R&D to production, distribution, and regulation. WHO advocates the promotion of R&D and accessibility of health products bolstering collaboration with pivotal stakeholders, including the United Nations system and other international organizations, academic and research institutions, civil society, and the private sector [3]. These entities are highly attentive to the challenges faced in health product R&D and accessibility, and have taken a series of actions to promote the rapid development of safer, more effective, and more affordable vaccines, medicines, medical devices and equipment. These efforts have paid off and have significantly reduced the disease burden worldwide, but mostly in high-income countries [4].

However, structural contradictions do exist in the supply and demand of health products in the Association of Southeast Asian Nations (ASEAN). For example, ASEAN countries are in the midst of strengthening their R&D systems, enhancing research capabilities, expanding production facilities, and cultivating competitiveness in locally made products, paving the way to provide an adequate supply of safe, effective, and affordable health products [5], and are at the same time dealing with demand challenges arising from multiple health needs, including emerging and re-emerging communicable diseases and non-communicable diseases [6]. In particular, residents in some remote and low-income areas struggle to access essential health services and pharmaceutical products, often relying on expensive imported products, which further exacerbates the economic burden and severely impedes the progress toward universal health coverage [7]. China and the ASEAN countries are in geographical proximity and share cultural similarities. Since the establishment of the China-ASEAN more than 30 years ago, cooperation in public health, including infectious disease prevention and control, and public health emergency preparedness and response, has enhanced the regional health security and promoted the well-being of people of the both sides. Promoting R&D and accessibility of health products has become a commonly shared public health priority for both China and ASEAN countries [8]. Effectively addressing the challenges of insufficient key technologies and production capacity for health products, so as to better meet the growing needs of local residents, has become one of the core tasks for the development of health industry in both China and ASEAN countries. Againt such a backdrop, regional actions to complement, leverage and maximize national efforts to promote access to essential health products and technologies are more important than ever before, and a broader and deeper cooperation between China and the ASEAN countries in this space has been building momentum and holds great promise.

## Recommended actions to promoting innovation based on forum consensus

Upholding the idea of promoting technology-led development with a focus on a cooperative solution, the China-ASEAN Science and Technology Cooperation Center for Public Health is building up a hub of research and development, technological innovation, and capacity building in public health between China and the ASEAN countries.

Our team initiated with an in-depth investigation was initiated into the subject matter and research has been conducted on the general concept, norms, prospects, principles of innovation and accessibility of health products. As a follow-up step, focus group and nominal group techniques were employed to gather opinions and facilitate open dialogues with the Center’s academic committee and other experts. It is believed that  this approach was instrumental in synthesizing diverse critical points and reaching a consensus, thereby ensuring a comprehensive integration of various perspectives. Furthermore, when the Center held the Second Annual Forum on China-ASEAN Cooperation in Public Health on June 26–28, 2024, the theme of innovations in development cooperation for medical devices and technology was explored among more than 200 experts from government departments and academia from China, Cambodia, Indonesia, Laos, Myanmar, Thailand, Vietnam, Singapore, Brunei Darussalam, as well as WHO and other international organizations. The attendees shared views on key issues in promoting the R&D and accessibility of health products, which greatly promoted high-level exchanges regarding science and technology cooperation in public health. The participants at the Forum reached consensus on launching the China-ASEAN Beijing Declaration on Cooperation in Innovation of Health Products and Technologies, which calls on China and ASEAN countries to carry out the following five actions:

1. Establish a more effective, open, and inclusive cooperation mechanism for health product innovation towards a China-ASEAN innovation ecosystem

We advocate for maximizing China and the ASEAN’s development potential and innovative vitality by linking enterprises, research institutions, and university research platforms. The objective is to establish a more effective, open, and inclusive cooperation mechanism for health product innovation to promote knowledge sharing, information exchange, collaborative research, and capacity building. Timely, continuous, accurate, and high-quality products, technology, and human resources will be provided through the mechanism for building a regional health product innovation ecosystem. Additionally, institutional and financial support will also be furnished through the mechanism for the entire R&D, production, and regulation chain.

2. Enhance innovative R&D capabilities by targeting new technologies, methods and appropriate and affordable health products for key populations and addressing diseases prevalent in China and ASEAN

We advocate for promoting R&D work guided by translational and evidence-based medicine to ensure robust clinical practice and data support in terms of effectiveness and safety. We call for the utilization of new technologies and methods, such as telemedicine systems, artificial intelligence, and big data, to enhance R&D efficiency and production capacity. We reiterate the importance of tackling dual imperatives: addressing the domestic concerns of individual countries, and simultaneously confronting shared health challenges across the region by developing appropriate and affordable health products for key populations and diseases. We call for the evidence-based formulation of R&D plans, optimization of R&D priorities, and the provision of new concepts, models, and systematic solutions.

3. Establish a China-ASEAN Collaboration Center for Health Product Innovation, coordinate regional development plans, and enhance equitable access to pharmaceutical products at regional level

We advocate for establishing a China-ASEAN Regional Center for Health Product Innovation based on the WHO's guiding framework. The Center will serve to ensure that the product development lifecycle, including R&D, production, distribution, and regulation, will comply with the WHO and related international norms and standards. We propose developing diversified market entry strategies and market cooperation plans, including sharing the latest research outcomes and best practices, along with relevant rules and standards, to enhance equitable access to pharmaceutical products in the region.

4. Accelerate regulatory harmonization in the region by optimizing and improving regulatory modalities of China and ASEAN Countries

We advocate for optimizing and improving the regulatory modalities for health products in the region. We will actively promote the coordination of regional and national development strategies and systems, exploring inter-country regulatory reliance and convergence, and thus driving the vigorous development of regulatory harmonization. We aim to scientifically balance intellectual property protection with the promotion of innovation, leverage market-shaping strategies, enhance cooperative trust, and achieve the dual goals of promoting R&D and access to health products.

5. Strengthen cross-sectoral cooperation to build resilient health system and achieve sustainable development of innovation cooperation

We advocate for integrating health into all policies through cross-sectoral cooperation. We advocate the establishment of coordination mechanisms between the health sector and related departments, such as regulation, financing, trade, and transportation to ensure the effective implementation of health product R&D and promotion of accessibility at both regional and national levels. We call for building resilient health systems, including establishing sustainable health financing strategies, enhancing the capacity of health workforce, and providing integrated healthcare services through primary health care. The resilient health system should be deeply integrated with the health product lifecycle to achieve sustainable development in innovation cooperation that promotes the R&D and accessibility of health products.

**Fig. 1:**
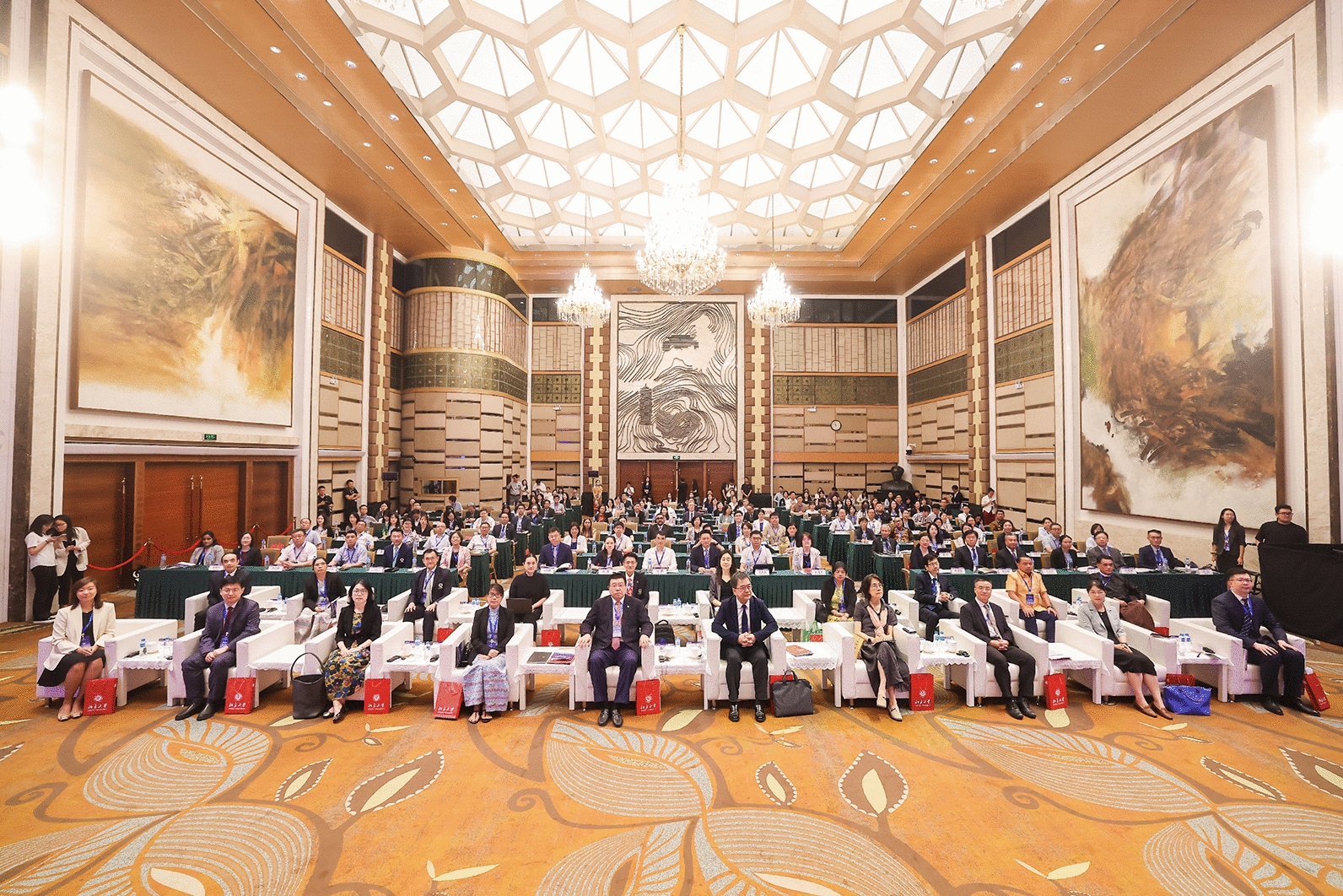
The second annual forum on China-ASEAN cooperation in public health on June 26-28, 2024 at Peking University

**Fig. 2:**
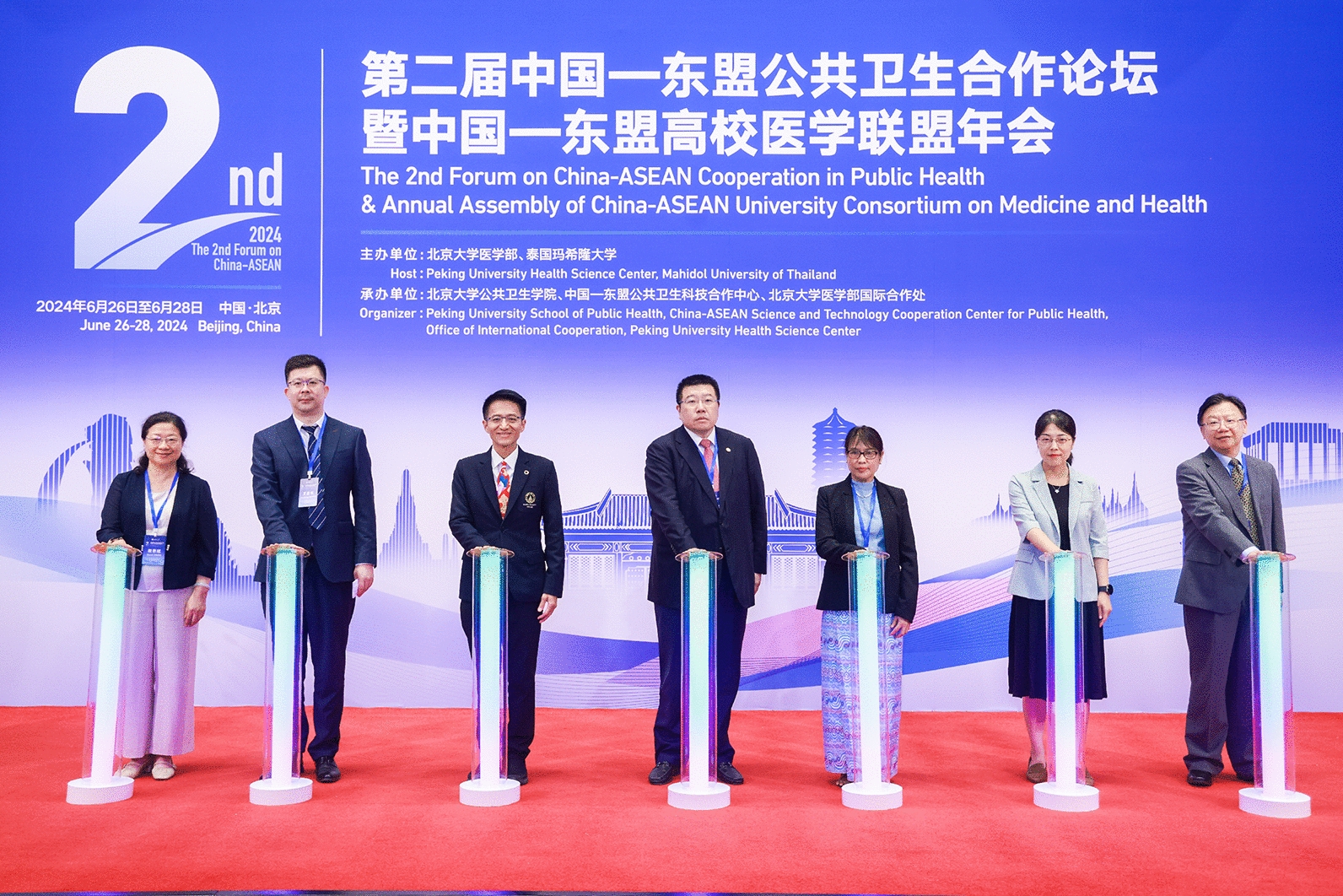
The launch of the China-ASEAN Beijing Declaration on Cooperation in Innovation of Health Products and Technologies on June 26, 2024

In light of this declaration, we pledge to join forces with our ASEAN partners to create opportunities for a stronger partnership. We will play a more active role in regional public health governance and development cooperation, continuously tackling the challenges in R&D and accessibility of health products, and addressing more complex public health system challenges. We will redouble our efforts to achieve the health-related sustainable development goals (Figures 1 & 2).

**Table:** 

Actions	Goals	Key Action
Action #1	Cooperation Mechanism	Knowledge sharing Information exchange Collaborative research Capacity building
Action #2	Innovation Capability	Evidence-based R&D plans formulation R&D priorities optimization New concepts, models, and systematic solutions adaptation
Action #3	Equitable Access	Market entry strategies Market cooperation plans Upholding WHO and related international norms
Action #4	Regulatory Harmonization	Regulatory reliance and convergence Intellectual property protection Innovation incentives
Action #5	Cross-sectoral Cooperation	Sustainable health financing strategies Improved Health workforce capacity Integrated health services through primary health care

## Data Availability

Data sharing not applicable to this article as no datasets were generated or analysed during the current study.

## Abbreviations

R&D: Research and Development
WHO: World health organization
ASEAN: Association of Southeast Asian Nations
